# The Association of Urban Greenness and Walking Behavior: Using Google Street View and Deep Learning Techniques to Estimate Residents’ Exposure to Urban Greenness

**DOI:** 10.3390/ijerph15081576

**Published:** 2018-07-25

**Authors:** Yi Lu

**Affiliations:** 1 Department of Architecture and Civil Engineering, City University of Hong Kong, Hong Kong, China; yilu24@cityu.edu.hk; 2 City University of Hong Kong Shenzhen Research Institute, Shenzhen 518057, China

**Keywords:** urban greenness, eye-level greenness, street greenness, physical activity, walking

## Abstract

Many studies have established that urban greenness is associated with better health outcomes. Yet most studies assess urban greenness with overhead-view measures, such as park area or tree count, which often differs from the amount of greenness perceived by a person at eye-level on the ground. Furthermore, those studies are often criticized for the limitation of residential self-selection bias. In this study, urban greenness was extracted and assessed from profile view of streetscape images by Google Street View (GSV), in conjunction with deep learning techniques. We also explored a unique research opportunity arising in a citywide residential reallocation scheme of Hong Kong to reduce residential self-selection bias. Two multilevel regression analyses were conducted to examine the relationships between urban greenness and (1) the odds of walking for 24,773 public housing residents in Hong Kong, (2) total walking time of 1994 residents, while controlling for potential confounders. The results suggested that eye-level greenness was significantly related to higher odds of walking and longer walking time in both 400 m and 800 m buffers. Distance to the closest Mass Transit Rail (MTR) station was also associated with higher odds of walking. Number of shops was related to higher odds of walking in the 800 m buffer, but not in 400 m. Eye-level greenness, assessed by GSV images and deep learning techniques, can effectively estimate residents’ daily exposure to urban greenness, which is in turn associated with their walking behavior. Our findings apply to the entire public housing residents in Hong Kong, because of the large sample size.

## 1. Background

According to the biophilia hypothesis, people possess a genetically-based tendency to affiliate with nature [1]. Indeed, recent studies have established that urban residents living in the neighborhoods with higher amount of urban greenness, comprising of parks, landscaped streets and open greenspaces, tend to have better health outcomes, such as reduced long-term stress [2], increased recovery speed after surgery [3], improved mood [4], healthier weight outcomes [5], lower risk of chronic diseases [6], and enhanced health-related quality of life [7].

Though the health benefits of urban greenness have been well-documented, the causal mechanisms are less clear. It has been suggested that exposure to urban greenness may link to physical and psychological benefits through different intermediate effects: through facilitating social cohesion of a community; through promoting physical activities with a supportive environment, such as cycling, walking, and green exercise; and by reducing exposure to air pollution, heat and noise [8,9]. The intermediate effect of physical activity has received research attention because conducting physical activity while exposed to greenness has synergistic benefits [10,11]. Physical activities performed in greenspaces can have greater health benefits than those performed in other environments [12]. Pretty and colleagues demonstrated significant blood pressure reduction and improved mood for adults with only five minutes of engagement in exercise in the presence of greenness compared with those who exercised in the absence of greenness [11,13]. 

The empirical studies investigating urban greenness-physical activity associations have so far delivered mixed results [14]. Many studies reported positive associations [15,16,17,18,19,20,21,22,23]. For example, the availability of street trees was positively associated with walking time [20]. Both the quantity and the quality of urban greenness, evaluated by a field audit, were significantly related to self-reported physical and psychological well-being [21]. Yet some studies reported that walking behavior was associated with subjectively assessed greenness but not objectively assessed greenness [24,25]. The inconsistence in the results may be explained by the fact that researchers have defined and measured urban greenness differently. Some population-level studies assessed urban greenness with park and tree count, or some standardized indexes from satellite imagery, e.g., normalized difference vegetation index (NDVI) [8,20,25]. Yet the amount of greenness measured by the number of parks or trees, NDVI or other overhead-view indexes often differs from the amount of greenness perceived by a person at eye-level on the ground, especially in locations with dense vegetations [26,27]. For instance, satellite imagery often fails to detect vegetation covered by urban canopy or vertical green walls. Therefore, overhead-view greenness measures may be inadequate for assessing people’s exposure to street greenness [26,27]. 

In addition, many studies focusing on urban greenness-physical activity associations have been justifiably criticized for their residential self-selection bias, which makes the impact of urban greenness on physical activity uncertain [28,29,30]. For example, people preferring walking may consciously choose to live in neighborhoods with a higher amount of greenness. Therefore, the observed urban greenness-physical activity associations can also be alternatively explained by intra-personal factors, instead of a true causal effect of the environment [30]. A research design implementing randomized controlled trials that experimentally assign residents to neighborhoods with different levels of greenness would be ideal for addressing this self-selection bias; however, it is politically impractical. 

Researchers have developed several alternative options to address this residential self-selection bias. Some studies have directly assessed individual preferences and attitudes and ruled them out in statistical models. For instance, Bagley and Mokhtarian [31] reported that the associations between walking and the built environment for residents from San Francisco, USA, were largely accounted for by personal attitudes and self-selecting into certain neighborhoods. Using data from Northern California, Handy, Cao and Mokhtarian [32] showed that the built environment still had an impact on walking after accounting for attitudes and preferences. Direct questioning, however, may suffer from recall bias or social desirability bias [33,34]. Some researchers have recommended longitudinal research design, with the assumption that individual attitudes and preferences are constant over time, therefore longitudinal studies can at least partially separate the effect of individual preference from the built environment–physical activity association [33,34]. Longitudinal research design often involves measuring physical activity before and after relocation or an environmental intervention [32,34]. Nevertheless, residential relocation is not randomly assigned to the participants. The change of travel behaviors may be alternatively explained by changes of job location and possible changes in lifestyle and attitudes toward physical activity associated with the relocations [33].

The current study addressed the abovementioned methodological limitations in two ways. First, we derived eye-level urban greenness from Google Street View (GSV) images, which is a readily available service providing eye-level streetscape images in many countries. The GSV views were captured by cars, trikes, or pedestrians moving along streets; we can access those images with a Python script working with the GSV API [35]. Those GSV images capture all types of vegetation along streets, difficult to be accurately assessed by other methods. Those images closely resemble the streetscape pedestrians perceive when traversing through urban environment. Therefore, people’s daily exposure to urban greenness can be more accurately assessed from those images. Several empirical studies have exploited GSV images to assess different features of urban environment [36,37,38,39]. Some previous studies primarily used color in images to identify vegetation from GSV images [27,40]. Yet, the color technique often falsely identifies man-made green objects, e.g., trucks, windows, or walls, as vegetation. Furthermore, recent advances in computer vision, particularly in deep learning, such as fully convolutional neural network (FCN), avoid this shortcoming by considering the shape of those objects as well, hence improving the accuracy. The deep learning techniques can segment an image into different parts and objects such as sky, vegetation, building, and road [41,42,43,44]. Pyramid scene parsing network (PSPNet) have achieved one of the best performances on the task of identifying vegetation from streetscape images; the pixelwise accuracy is as high as 93.4% [45]. In the present study, we used PSPNet to automatically detect the amount of street vegetation in GSV images [45]. 

Second, to reduce the residential self-selection bias inherent to most urban greenness-physical activity studies, we exploited the research opportunity arising in a citywide resident relocation scheme. Approximately two million low-income Hong Kong residents live in more than 170 public housing estates which are heavily subsidized by the government [46,47]. They were assigned to different housing estates largely according to family sizes and flat availability rather than their individual preferences for built environment characteristics. Therefore, the Hong Kong public housing scheme provides a promising situation to investigate the impact of the built environment on physical activity while significantly reducing the residential self-selection bias. Hong Kong public housing estates are also excellent foci for design intervention. The centralized land control and single ownership of a public housing estate allows for the simple introduction of environmental interventions, especially in comparison to what is possible for a neighborhood setting. Any potential design intervention can stimulate the physical activity of numerous residents living in public housing estates in Hong Kong. 

In the present study, the association of eye-level urban greenness and walking behavior was explored for the residents of public housing estates in Hong Kong, after controlling for other built environment and individual covariates. The present study focused on walking behavior due to the data availability. In addition, walking is the most popular habitual form of physical activity among adults because it can be done at any time, alone or in company, requiring no special skills or expensive equipment [48]. 

## 2. Methods

### 2.1. Walking Data

Hong Kong has a total of 7.29 million residents and a relatively small land area of only 1104 km^2^ [49]. It is a developed coastal city located in the southeast of China. Its subtropical climate is mild, and its streets typically feature evergreen vegetation.

We obtained the data of walking trips from the 2011 Hong Kong Travel Characteristics Survey (HKTCS). Detailed descriptions of HKTCS are available in Reference [40]. The HKTCS was commissioned by The Transportation Department to identify the general travel behaviors of all Hong Kong population, and thus has a large sample size. For the main survey, 24,773 participants living in public housing estates are spatially distributed throughout the city. Trained interviewers conducted face-to-face interviews to get personal information (e.g., age, gender, dwelling location, household income) and travel behaviors (number of trips, trip time, and mode choice) during the last 24 h. The survey response rate was 71%. From the main survey, we can identify participants who engaged in walking during the last 24 h. 

The interviewers conducted an additional survey for a subset of 1994 public housing residents engaging in walking at least once during the last 24 h to get walking time for all walking trips. Therefore, we can obtain the total walking time (in minutes) for those 1994 participants. Ethical approval for the study was obtained from the Research Committee of City University of Hong Kong (H000691).

### 2.2. Street Greenness

The eye-level street greenness was derived from Google Street View (GSV) images using the PSPNet technique [45]. Using the reported dwelling address, participants’ dwelling location were geocoded in a digital map with ArcGIS 10.5 (Esri, Redlands, CA, USA). Currently, there is no consensus on the definition of neighborhoods, which were often operationalized in three different ways depending on data sources: administrative/census areas, a distance buffer around participants’ dwelling locations, and a self-perceived area with a 10–20 min walk from home [50]. The 400 m and 800 m distances take approximate 5 and 10 min to cover respectively, with a typical walking speed of 80 m/min [51]. Therefore, we also choose the 400 m and 800 m circular buffers of participants’ dwelling locations as neighborhood boundaries, which is in line with studies using objective measures [52,53,54,55]. Two buffers were used to mitigate the modifiable area unit problem (MAUP), which is the statistical bias that physical activity-built environment associations are influenced by the scale of the aggregation unit [56,57]. The potential greenness-walking association will prove robust if it remains significant across two different neighborhoods boundaries.

**Figure 1:**
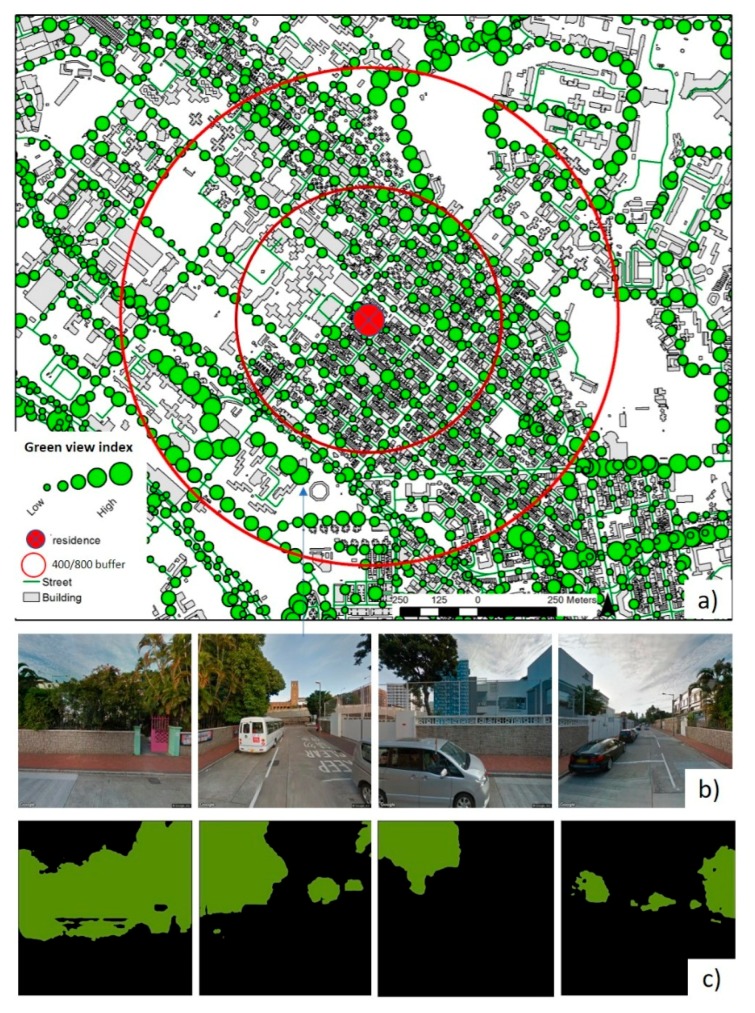
Eye-level street greenness assessment with PSPNet, a computer deep learning technique. (**a**) Green view index of the 400/800 m buffer around a dwelling location. Sampling points with 50 m spacing were generated in the street centerlines. (**b**) With a Python script developed by us, we can retrieve four streetscape images with a 90-degree field of view for a point. (**c**) All street vegetation in images were segmented with PSPNet.

Sampling points were generated in the street centerlines with a 50 m spacing in the buffers (Figure 1b). With a Python script we developed, we can retrieve four streetscape images with a 90-degree field of view for a point (Figure 1b). We used the PSPNet trained on the cityscape dataset, a repository of 5000 streetscape images from 50 cities with pixel-level annotations [58]. The trained model achieved a remarkable pixel-level accuracy of 93.4% in terms of identifying vegetation on the cityscape dataset [59]. With the PSPNet greenness extraction function in the script (Figure 1c), the amount of greenness for each point can be determined by the green view index—the proportion of greenery pixels in four images—as shown in the following equation [27]:

(1) $$\mathrm{Green}\;\mathrm{view}\;\mathrm{index}\;=\;\frac{\sum_{i\;=\;1}^{4}Greenery\;{pixels}_{i}}{\sum_{i\;=\;1}^{4}Total\;{pixels}_{i}}$$

Green view index values range between 0.0 and 1.0, with higher values representing higher levels of eye-level greenness (Figure 1a). The average green view index for all points in a buffer was used to assess the neighborhood around a dwelling location. To validate the PSPNet greenness extraction, vegetation was manually selected by an expert using Adobe Photoshop for 50 images. The pixels representing vegetation in each image was selected using the magic wand tool and adjusted with the lasso tool in Photoshop CS6 (Adobe, San Jose, CA, USA). The selected pixels were then counted in Photoshop and green view index was calculated again for expert judgement. The amount of street greenness extracted by PSPNet and expert judgement were strongly correlated, r(48) = 0.91; *p* < 0.01. Our validation demonstrated the reliability of GSV greenness extraction.

### 2.3. Covariates

Other built environment characteristics were also included in this study because of their potential influences on walking behaviors. Street intersection density [60,61,62], land-use mix [63], population density [60], number of shops, distance to the closest Mass Transit Rail (MTR) station, and number of bus stops [64,65] were objectively assessed in the buffers of participants’ dwelling locations in GIS platform. The land-use mix was assessed by entropy score to show the degree of land use diversity of three types: Commercial, office, and residential [63]. The personal information—including age, gender and household income—were extracted from the HKTCS survey and included in the study. 

## 3. Data Analysis

Walking behaviors were measured two ways in HKTCS: The decision of walking or not for 24,773 public housing participants, and the walking time of a subgroup of 1994 participants who walked at least once during the last 24 h. Correspondingly, two separate multilevel modeling were conducted. In analysis 1, we used logistic regression to examine the associations of street greenness with the likelihoods of walking. In analysis 2, we used linear regression to examine the association of street greenness and walking time. The monthly household income data were originally coded into 16 bands, and were converted to 4 bands (<15,000, 15,000–25,000, 25,000–50,000, and >50,000 HKD). Age was converted to a 4-band variable (2 to 17 years, 18 to 44 years, 45 to 64 years, and ≥65 years).

In both analyses, multilevel modeling was used to explain the clustering pattern of the walking behaviors for participants from the same urban area. The urban areas were defined as street blocks, a census unit with one or several housing estates and homogeneous socio-economic status. In both analyses, continuous variables were standardized as z-score transformation.

The multilevel modeling was conducted in R [66] with the ‘lme4’ package. Odds ratios (OR), 95% confidence intervals (CI), and standardized β were reported for the modeling fitting. Before modeling, careful attention was paid to the correlations among predictors. The Variance Inflation Factors (VIFs) were checked in R with the ‘usdm’ package. All VIFs were low (<2), indicating that multicollinearity was not present [67,68].

## 4. Results

The descriptive statistics of the participants were shown in Table 1. The female participants slightly outnumbered the male ones in Analysis 1 and 2. A large proportion of participants had medium-low household income (42.3% in analysis 1, and 40.8% in analysis 2); our participants had lower income than the Hong Kong population average because they were public housing residents. The sample in Analysis 2 only includes the participants who had at least one walking trip. Therefore, the elderly and the female were oversampled in Analysis 2 than in Analysis 1, because the elderly and the female had higher odds of walking as shown in the results of Analysis 1 (Table 2). 

The logistic regression results of analysis 1 were shown in Table 2. Interclass correlation coefficient (ICC) for the null model predicting the odds of walking and total walking time was 7.9% and 16.0% respectively, indicating the respective proportion of total outcome variation that is attributed to differences between street block. 

The green view index was related to higher odds of walking in both buffers after adjusting for covariates (OR (95% CI): 1.149 (1.035, 1.276) in 400 m buffer, 1.193 (1.070, 1.330) in 800 buffer). One standard deviation increase of the green view index increases the likelihood of walking by 14.9% and 19.3% in the 400 m and the 800 m buffers respectively.

Among other built environment factors, distance to MTR station was related to higher odds of walking in both buffers. Number of shops was positively related to higher odds of walking in the 800 m buffer, but not in the 400 m buffer. The associations of remaining built environment factors were insignificant. Among individual factors, female participants had higher odds of walking compared with their male counterparts. Participants in the medium-low, medium-high and high income group had lower odds of walking compared with those in the low income group. The result indicates that household income was negatively related with the walking decision. Age has a more complex relationship with the odds of walking. Adults (18–44, 45–65 years) had lower odds of walking and older adults (≥65 years) have higher odds, compared with children (5–17 years). The female participants had higher odds, compared with the male participants. The interaction term of the green view index*gender was significant in both buffers, indicating that there is a significant difference by gender in the association of the green view index and the odds of walking. Post-hoc analysis revealed that the association was stronger for females (1.176 (1.063, 1.301), *p* = 0.002 in 400 m, 1.235 (1.108, 1.378), *p* < 0.001 in 800 m) than for males (1.127 (1.002, 1.268), *p* = 0.046 in 400 m, 1.181 (1.048, 1.331), *p* = 0.006 in 800 m).

The linear regression results of analysis 2 were shown in Table 3. Eye-level greenness was associated with more walking time in both buffers; β (95% CI): 0.149 (0.045, 0.253) in the 400 m buffer, 0.233 (0.133, 0.333) in the 800 m buffer. Participants in the medium-low, medium-high and high income group had shorter walking time compared with those in the low income group. None of other built environment or individual factors was significantly related with total walking time. The interaction term of the green view index × gender was not significant in either buffer, indicating that there is no significant difference by gender in the association of green view index and total walking time for those walked at least once.

## 5. Discussion

### 5.1. Major Findings

Many empirical studies support the assertion that urban greenness has a variety of health benefits for urban residents. Yet explicit evidence has been inadequate for guiding design and planning decisions and policy to shape street landscape or greenspaces for long-term population health promotion [69]. Quantifying greenness-induced movement calls for the precise estimation of urban residents’ daily exposure to greenness as they move within a city [65,70]. This study is one of the first to quantitively assess eye-level greenness with advanced deep learning techniques and link it with walking behavior.

In the present study, we found that the eye-level greenness measured by GSV images was related to both higher odds of walking and longer walking time for 24,773 and 1994 public housing residents respectively. These are novel and robust findings given that this study engaged a large sample size, used multiple buffer sizes to define a neighborhood, and adopted a research design to reduce residential self-selection bias. Our results support that urban greenness has a beneficial effect on walking, supporting previous studies regarding the association between urban greenness and physical activity [9,71,72]. It is worth noting that this study highlighted the importance of landscaped streets in addition to parks and large open green spaces, because most GSV images were taken from streets. Green streets may facilitate walking behaviors by making walking routes attractive with beautiful street landscapes and by making walking routes comfortable with reduced heat, noise and air pollution. The availability of street vegetation improves aesthetical judgement for urban environment, which have been identified as key built environment factors of walking behaviors [70,73].

Our results are in accordance with the previous results that residents tend to be healthier living in greener urban neighborhoods. For instance, children had lower chances of having obesity and asthma [71,72]; older adults enjoyed greater longevity [74], general adults had better perceived general health conditions [3] in neighborhoods with more street vegetation. This study sheds light on the casual mechanism between the street greenness-health associations, by suggesting that increased walking is a mediating pathway.

By the same token, our findings contrast the non-significant or negative associations between urban greenness and physical activity [24,25,75]. It may be explained by the different ways of assessing urban greenness. Currently, most health studies use one of two methods to objectively assess the amount of urban greenness: Field audits [21,76], and Geographic Information System (GIS) [20,71,72,77]. Field audits are relatively time-consuming and inefficient because the observers need to physically visit all sites. GIS is objective and efficient; yet some street vegetation data, such as shrubs or lawns, were often not collected in GIS. In addition, GIS-based methods generally measure the availability of street greenness from an overhead view, which may significantly differ from the resident’s exposure to those greenness at eye level, especially in locations with dense greenness [26,27]. Hence, the GSV method more precisely estimates the resident’s exposure to vegetation in an urban neighborhood than other methods. Subjective greenness but not NDVI—an index of greenness based on remote sensing imagery in GIS—was positively related to walking behaviors of 529 participants in Seattle, Washington [25], suggesting residents’ daily exposure to and perception of urban greenness may not be totally captured by GIS. Therefore, using GSV to quantitively assess eye-level greenness may be an efficient and innovative way to measure the people’s exposure to urban greenness.

It is worth noting that our participants are mostly low-income individuals because this study focuses on public housing residents. Household income is demonstrated to be negatively related to the odds of walking and total walking time; i.e., the poorer participants walk more than the wealthier participants (Table 2 and Table 3). The results also show that the distance to MTR station was positively associated to the likelihood of walking in both buffers (Table 2). Taking together, these results indicate that the public transportation system has a greater influence on the poorer individuals than affluent ones because poorer people often have no alternative transportation options.

Our results also show that the objectively measured 3D’s of the built environments (population density, land use mix, street intersection density) [73,78,79,80], were not related to decision of walking or walking time. Some recent studies from other high-density cities in South America and Asia have also demonstrated non-significant or contrary findings [54,81,82,83,84,85], compared with those reported in Western countries, especially the United States and Australia [73,78]. It suggests more complex relationships between the three D’s approach and walking or physical activity, which may be moderated by local built environment and social contexts.

This study also reveals that some individual factors were significantly associated with walking behavior. Female participants had higher odds of walking than male participants. The association of the green view index and the odds of walking was also stronger for female participants than for male participants. For those who walked at least once during the reference 24 h, gender is not associated with total walking time, and there is no significant difference by gender in the association of green view index and total walking time. Among all age groups, older adults (≥65 years) have the highest odds of walking, followed by children (5–17 years), then adults (18–44, 45–65 years). Older Chinese adults may pay more attention to their personal health for cultural reasons. Household income was negatively associated with both the odds of walking and total walking time. Family member of wealthier household may rely less on the public transportation system, therefore walking less.

The evidence from this study will help government agency develop targeted interventions in the form of urban planning to promote walking and the general health of residents in Hong Kong. First, urban planners should consider the location and visibility of urban greenness to make it effectively exposed to residents. Second, they should also pay close attention to the needs and travel behaviors of poor residents when making design decisions about public transportation infrastructure (e.g., availability and proximity of MTR stations) because those residents heavily rely on the public transportation system. Third, contrary to the suggestions for low-density Western cities, increasing urban density, street connectivity or land-use mix may be ineffective to promote walking in high-density cities, such as Hong Kong.

### 5.2. Strength and Limitation

The availability of the GSV dataset, coupled with recent advance in deep learning techniques, provides a unique opportunity to estimate resident’s daily exposure to urban greenness, which in turn sheds lights on the understanding of urban greenness’s impact on physical activity and health outcomes. Such advances can help us develop critical evidence for urban planner and policymakers to make informed decisions about how to design or reshape urban greenness to improve urban residents’ wellbeing. Additionally, this study exploited a citywide public housing scheme to reduce self-selection bias, identified as the primary limitation in built environment-health research [28,29,30]. Hence, positive relationships between urban greenness and walking behaviors observed in this study can be largely attributed to the effect of the environment on physical activity, rather than residential choice. Furthermore, the walking data were extracted from a population-level survey; the large sampling size warranted the reliability of our findings.

The study also has several limitations. Though this study reduced residential self-selection bias, we still cannot make any causal inference because of the cross-sectional research design adopted in this study. Longitudinal studies collecting data over multiple time points are warranted to address this issue. The factors of greenery exposure and MTR proximity may be correlated. The areas close to MTR stations often feature higher urban density and lower green view index than the areas far away from MTR stations. It is plausible that a longer walk from or to the MTR station is more likely to result in greater greenery exposure. Yet, walking routes were not reported by our participants, therefore we cannot test this assumption. The walking data were self-reported and were thus subject to recall bias. Participants may underreport short walking trips, especially for those living in dense urban environment. The walking and other physical activity behaviors can be objectively collected in future studies, such as accelerometers and GPS devices. The neighborhoods boundaries were defined using circular buffers rather than street network buffers of participants’ dwelling location, because some information of pedestrian infrastructure was unavailable yet, such as footbridges, elevated walkways, or corridors passing through buildings, which are common in the dense urban environment in Hong Kong. Further studies with detailed data of pedestrian infrastructure may consider using network buffers instead. Safety is also one of important factors that is positively associated with walking [73,86]. Yet safety-related data, such as traffic incidents or crime rates were currently unavailable. Further studies may incorporate safety-related data in the analysis. Some limitations stem from the GSV service. Some cities and districts are not covered by GSV. Thus the streetscape images were not accessible for those areas [35]. GSV images were often taken by cameras installed on top of vehicles moving along streets, hence those images may slightly differ from what pedestrians see while walking along sidewalks.

## 6. Conclusions

This study demonstrates that eye-level greenness is positively associated with the odds of walking and walking time for public housing residents in Hong Kong. Eye-level street greenness assessed by GSV, in conjunction with deep learning techniques, can accurately and effectively estimate people’s exposure to urban greenness, compared with existing methods. Therefore, it can contribute to methodological development of health studies. The findings of this study also have some implicit planning applications. Governments and urban planners should consider not only the provision of urban greenness in terms of general density or size, but also the visibility of the greenness from a pedestrian’s perspective while moving through a city.

## Figures and Tables

**Table 1:** Characteristics of study Participants. (Hong Kong SAR, China in 2011. *n* = 24,773 in Analysis 1, *n* = 1994 in Analysis 2).

Sociodemographic Variables	Analysis 1 (*n* = 24,773)		Analysis 2 (*n* = 1994)	
	Count	Percentage (%)	Count	Percentage (%)
Age				
5–17	3770	15.2	337	17.2
18–44	9456	38.2	583	29.8
45–64	7905	31.9	646	33
≥65	3642	14.7	392	20
Gender				
Male	11,924	48.1	852	43.5
Female	12,849	51.9	1106	56.5
Household income				
Low (<10 k HKD)	6231	25.2	583	29.8
Medium-low (10–20 k)	10,471	42.3	798	40.8
Medium-high (20–30 k)	5655	22.8	445	22.7
High (>30 k)	2416	9.8	132	6.7

**Table 2:** Multilevel logistic regression analysis for the relationship between greenness and built environment and individual factors, and the odds of walking in analysis 1; *n* = 24,773.

Model Predictors	400 m Buffer	*p*-Value	800 m Buffer	*p*-Value
	OR, (95% CI)		OR, (95% CI)	
**Greenness**				
Green view index	1.149, (1.035, 1.276)	0.009 *	1.193, (1.070, 1.330)	0.001 *
**Built environment**				
Population density	1.050, (0.957, 1.152)	0.304	1.047, (0.955, 1.148)	0.329
Land-use mix	1.039, (0.959, 1.126)	0.354	1.020, (0.935, 1.111)	0.659
Intersection density	1.031, (0.932, 1.140)	0.556	1.003, (0.859, 1.172)	0.967
Number of retail shops	1.056, (0.962, 1.160)	0.252	1.191, (1.049, 1.353)	0.007 *
Number of recreational facilities	1.008, (0.924, 1.099)	0.859	1.000, (0.884, 1.132)	0.996
Number of bus stops	0.997, (0.903, 1.101)	0.950	0.948, (0.804, 1.119)	0.529
Distance to MTR	1.090, (1.027, 1.156)	0.005 *	1.095, (1.025, 1.169)	0.007 *
**Individual factors**				
Age				
5–17—Reference				
18–44	0.354, (0.327, 0.383)	<0.001 **	0.354, (0.326, 0.383)	<0.001 **
45–64	0.551, (0.507, 0.594)	<0.001 **	0.551, (0.506, 0.598)	<0.001 **
≥65	1.763, (1.590, 1.950)	<0.001 **	1.760, (1.593, 1.950)	<0.001 **
Gender				
Male—Reference				
Female	1.585, (1.501, 1.672)	<0.001 **	1.585, (1.501, 1.672)	<0.001 **
Household income				
Low (<10 k)—Reference				
Medium-low (10–20 k)	0.806, (0.751, 0.865)	<0.001 **	0.806, (0.751, 0.865)	<0.001 **
Medium-high (20–30 k)	0.675, (0.621, 0.733)	<0.001 **	0.675, (0.622, 0.734)	<0.001 **
High (>30 k)	0.555, (0.498, 0.620)	<0.001 **	0.554, (0.497, 0.618)	<0.001 **
**Interaction term**				
Green view index × Gender	1.070, (1.014, 1.129)	0.014 *	1.091, (1.034, 1.152)	0.001 *
**Model fitting**	AIC = 31025 BIC = 31204 −2 Log Likelihood = −15,490		AIC = 31015 BIC = 31193 −2 Log Likelihood = −15,485	

Note: *p* < 0.05 *, *p* < 0.001 **. All model predictors (greenness, built environment and individual factors) were entered into models simultaneously.

**Table 3:** Multilevel linear regression analysis for the relationship between greenness and built environment and individual factors, and walking time (in minutes) in level 2; *n* = 1994.

Model Predictors	400 m Buffer	*p*-Value	800 m Buffer	*p*-Value
	β, (95% CI)		β, (95% CI)	
**Greenness**				
Green view index	0.149, (0.045, 0.253)	0.005 *	0.233, (0.133, 0.333)	<0.001 **
**Built environment**				
Population density	0.007, (−0.083, 0.097)	0.875	−0.042, (−0.129, 0.044)	0.337
Land-use mix	0.048, (−0.036, 0.133)	0.261	0.006, (−0.083, 0.094)	0.900
Intersection density	0.055, (−0.047, 0.157)	0.287	0.133, (−0.021, 0.287)	0.090
Number of retail shops	−0.017, (−0.116, 0.081)	0.730	0.022, (−0.103, 0.146)	0.734
Number of recreational facilities	0.017, (−0.072, 0.106)	0.704	−0.100, (−0.210, 0.011)	0.076
Number of bus stops	0.061, (−0.041, 0.164)	0.241	0.068, (−0.086, 0.221)	0.384
Distance to MTR	−0.004, (−0.074, 0.066)	0.910	0.012, (−0.065, 0.089)	0.753
**Individual factors**				
Age				
5–17—Reference				
18–45	−0.021, (−0.144, 0.102)	0.742	−0.022, (−0.143, 0.105)	0.758
45–64	0.097, (−0.023, 0.221)	0.114	0.101, (−0.020, 0.223)	0.101
≥65	0.043, (−0.101, 0.189)	0.548	0.057, (−0.086, 0.201)	0.430
Gender				
Male—Reference				
Female	0.057, (−0.026, 0.140)	0.180	0.056, (−0.027, 0.139)	0.189
Household income				
Low (<10 k)—Reference				
Medium-low (10–20 k)	−0.110, (−0.220, 0.000)	0.050 *	−0.120, (−0.229, −0.010)	0.032 *
Medium-high (20–30 k)	−0.245, (−0.372, −0.119)	<0.001 **	−0.242, (−0.368, −0.116)	<0.001 **
High (>30 k)	−0.365, (−0.554, −0.177)	<0.001 **	−0.376, (−0.564, −0.188)	<0.001 **
**Interaction term**				
Green view index × Gender	0.072, (−0.012, 0.156)	0.093	0.075, (−0.010, 0.160)	0.085
**Model fitting**	AIC = 5502 BIC = 5636 −2 Log Likelihood = −2727		AIC = 5481 BIC = 5614 −2 Log Likelihood = −2716	

Note: *p* < 0.05 *, *p* < 0.001 **. All model predictors (greenness, built environment and individual factors) were entered into models simultaneously.
